# Cases in Practice

**Published:** 1865-09

**Authors:** Walter Hay

**Affiliations:** Chicago, Ill.


THE
### CHICAGO MEDICAL EXAMINER.

N. S. DAVIS, M.D., EDITOR.

VOL. VI. SEPTEMBER, 1865. NO. 9.

### Original Contributions.

### ARTICLE XXVIII.

### CASES IN PRACTICE.

BY WALTER HAY, M.D., Chicago, Ill.

As the opinions of some of the latest pathological writers, upon the nature, causes, and appropriate treatment of that often occurring phenomenon, inflammation, and its neuro-pathic relations, appear to be undergoing important modifications, under the additional light thrown upon the subject, by the advances made in the study of the physiology of the nervous system, any facts, however trivial, bearing upon the subject, may be not unimportant or uninteresting.

The three subjoined cases have been selected from a number, as illustrating more clearly the principle which is believed to be involved in their history, viz., that inflammatory action is in many, if not all cases, the result of a paretic condition of the nervous system to a greater or less degree, most probably in all cases, of the vaso-motor system, consequently of diminished rather than increased vitality, and, therefore, requiring a sustaining and even stimulating rather than an antiphlogistic treatment.

The evidence furnished, also, in corroboration of the experiments quoted below, to determine the function of the sympathetic nerve, may have some value; and, while far from sufficient to establish a principle, or even to furnish the basis for a theory, in itself, may yet suggest further observation of similar phenomena, and the collation of additional facts bearing upon the same subject, a very wide field of pathology being open to the experiment.

The value of alkaloid strychnia, in alleviating certain forms of pain, scarcely needs additional evidence, but can be explained in no way so satisfactorily as, that pain is also a manifestation of nervous paresis.

CASE I. Mrs. F., aged about 45 years, of unusually robust constitution, free from any recognizable hereditary or acquired taint, was attacked, in March, 1861, with acute sclerotico-conjunctivitis in the right eye, the conjunctiva becoming rapidly ulcerated, accompanied by intense pain, effectually preventing sleep, and great photophobia, with considerable febrile reaction. The only cause to which this action could possibly be attributed, after very careful investigation, was fatigue.

The usual treatment, viz., cooling and anodyne applications, counter-irritation, by vesication of the mastoid and temporal region, and mercurial alteratives, was adopted, which was followed by *recovery* in about four weeks.

This condition was reproduced in the same patient, in November, 1863, following, as in the former case, *excessive fatigue.* The case came into my hands, on this occasion, after having been treated by a skilful oculist for nine weeks, and *recovered,* after an additional period of two weeks, under the mode of treatment used on the former occasion.

In March, 1864, the same patient was subjected to a third attack from, apparently, the same cause. On this occasion, the inflammation was more severe than formerly, the mucous membrane becoming ulcerated within thirty-six hours. The patient was entirely prevented from sleeping, during two nights, from excessive pain. On the third day from the beginning of the attack, the right eye was completely ecchymosed, presenting over the centre of the cornea a vertical ulcer, four lines by one in its dimensions.

Believing this condition to result solely from nervous exhaustion, and to be analagous in character, though different, of course, in degree from the results observed 1732, by POURFOUR DU PETIT, after the division of the trunk of the sympathetic, opposite the fourth or fifth cervical vertebra in dogs, viz., "great disturbance in the circulation of the eyeball, producing inflammation, &c., &c., and, ultimately, ulceration and destruction of the entire organ."

DUPUY reports, in *Journal de Medicine, Chirurgie, &c., Decembre*, 1816, that, after extirpation of the superior cervical ganglion, the same local results, with regard to the eye, followed.

Dr. JNO. REID reports, (*Edinburgh Medical and Surgical Journal, August*, 1839,) that the conjunctiva became red and congested in a few minutes after cutting and irritating the sympathetic nerve in the neck. The observations of many other physiologists might be cited, were the above insufficient, to establish the dependence of the histogenetic process, upon the integrity of the (sympathetic) ganglionic nervous system.

Admitting, then, the results of the above observations as correct, it would appear, not an unreasonable inference, that the diseased condition of the eye, in the case which I have cited, was due to interference with the influence of the ganglionic nervous system upon the diseased organ, and that interference in this case was the result of a paretic condition of a portion of that system, most probably of the ciliary ganglion.

With this impression of the case in mind, I directed the patient, on the third day from the commencement of the attack, no other treatment having yet been adopted, to take one-sixteenth of a grain of strychnia every four hours. After having taken four doses, the patient was free from pain, and slept well for eight hours, at which time there was a marked improvement in the condition of the eye, which continued until sixteen doses had been taken, after which all treatment was abandoned. There being, with the exception of a narrow cicatrix upon the site of the ulcer, no difference in the appearance of the two eyes. The length of time elapsing from the beginning of the attack, until complete recovery, being seven days, and from the commencement of the treatment four days.

Case II. *May*, 1864. E. K., a little girl, aged eight years, of decidedly strumous diathesis, having had, during the previous autumn, a very extensive eruption of favus, was seized with rigors, followed by febrile reaction; pulse averaging about 120, small and feeble, with great lassitude, followed by sore mouth. When seen for the first time, on the eighth day, since the onset of the fever, she was in the condition above described; there was a free flow of saliva, the edges of the tongue, gingival and buccal surfaces presenting numerous ulcerations, one of which was as large as a dime, and, by reason of the swelling of the surrounding surface, a line in depth; there was some diarrhœa, with slight tenderness upon pressure over the abdomen. The only food that could be taken was milk, in small quantities. Directed one-fiftieth grain of strychnia to be given every four hours. On the fifth day, the child was so far recovered as to be able to eat the crust of bread, and the treatment was discontinued, as no longer necessary.

Case III. Ulcerative stomatitis, in a child, four years of age, a patient in St. Luke's Hospital. In this case one-fiftieth grain of strychnia was administered every six hours, and was followed by entire recovery in four days.

These three cases, occurring at wide intervals of space, and in patients essentially differing in constitutional peculiarities, social position, and surroundings, while the morbid phenomena even are manifested in totally different organs, present, however, one pathological element, and that one the most prominent, viz., ulceration of mucous membrane, in common; there was, moreover, in each case, a marked identity in its cause, exhaustion, in the first case fatigue; in the second, fatigue, together with exposure to the sun; in the third, exhaustion from imperfect nutrition and bad air, the child being a pauper. In the face of the evidence, presented by each case in its turn, enlightened by the results of the physiological experiments already quoted, the only reasonable diagnosis appeared to be paresis of the sympathetic nervous system, and this diagnosis, together with the very great value of strychnia as a nervous tonic, has, apparently, been demonstrated by the results.

It is hoped that further experiments will be made and reported, either in confirmation or refutation of the views herein offered, as the subject offers many opportunities to observers.

